# How good is an explanation?

**DOI:** 10.1007/s11229-022-04025-x

**Published:** 2023-02-02

**Authors:** David H. Glass

**Affiliations:** School of Computing, Ulster University, York St, Belfast, BT15 1ED UK

**Keywords:** Explanation, Explanatory goodness, Explanatory power, Bayesian, Information, Confirmation

## Abstract

How good is an explanation and when is one explanation better than another? In this paper, I address these questions by exploring probabilistic measures of explanatory power in order to defend a particular Bayesian account of explanatory goodness. Critical to this discussion is a distinction between weak and strong measures of explanatory power due to Good (Br J Philos Sci 19:123–143, 1968). In particular, I argue that if one is interested in the overall goodness of an explanation, an appropriate balance needs to be struck between the weak explanatory power and the complexity of a hypothesis. In light of this, I provide a new defence of a strong measure proposed by Good by providing new derivations of it, comparing it with other measures and exploring its connection with information, confirmation and explanatory virtues. Furthermore, Good really presented a family of strong measures, whereas I draw on a complexity criterion that favours a specific measure and hence provides a more precise way to quantify explanatory goodness.

## Introduction

It would be difficult to overstate the interest among philosophers of science on the topic of explanation. Much of this has focussed on the nature of explanation (Woodward, 2017), with modern discussions stemming from the deductive-nomological model of Hempel and Oppenheim (1948) and going on to consider other models such as statistical relevance (Salmon, 1971), unification (Friedman, 1974; Kitcher, 1989), causal-mechanical (Salmon, 1984), causal (Woodward, 2003) and pragmatic accounts (van Fraassen, 1980). Although not receiving so much attention, there has also been interest in quantifying or comparing explanations using probability (Popper, 1959; Good, 1960, 1968), including a number of recent proposals (Schupbach and Sprenger, 2011; Crupi and Tentori, 2012; Glass, 2007, 2021).

It might be thought that an answer to the question ‘what is an explanation?’ would be needed before attempting to answer questions such as ‘how good is an explanation?’ or ‘when is one explanation better than another?’, but that does not seem to be the case. Discussions about the nature of explanation typically involve pre-theoretical intuitions about explanation and often these extend to intuitions about the goodness of explanations, particularly comparative judgments. One can hold that quantum mechanics provides a very good explanation of blackbody radiation or that thermodynamics provides a better explanation of heat than the caloric theory without settling the question of what exactly constitutes an explanation. Similarly, in the area of medical diagnosis, one might try to formalize comparative judgments about which condition best explains the symptoms without taking a view on the metaphysics of explanation.

Explanatory goodness is clearly very important in the context of inference to the best explanation (IBE) (Lipton, 2004; Douven, 2017). While defenders of IBE need not be committed to a particular view on the nature of explanation, they nevertheless need to be able to give an account of the goodness of an explanation and more specifically how to compare explanations. In this context, discussion of explanatory virtues is important with explanations that do better according to a range of virtues being judged better (Mackonis, 2013). Of particular relevance here are approaches to IBE that seek to evaluate explanations using probability theory (Douven, 1999, 2013; Schupbach, 2018; Glass, 2012, 2021), though one might expect that such approaches should also capture at least some of the explanatory virtues. However, questions about explanatory goodness and comparative judgments, which arise both within science and outside it, are still important irrespective of whether one is committed to IBE as a legitimate mode of scientific inference.

In this paper, I will focus on measures of explanatory power to address the central question. A variety of probabilistic measures of explanatory power in this sense have been proposed in the literature (Good, 1968; Schupbach and Sprenger, 2011; Schupbach, 2011, 2018; Crupi and Tentori, 2012). Arguably, these measures can be seen as attempts to quantify how well a hypothesis would explain a given explanandum *if the hypothesis were true*. According to a distinction due to Good (1968), which is central to the current paper, these are measures of *weak explanatory power* whereas strong measures take into account not only how well the hypothesis would account for the explanandum if it were true, but also *how likely it is to be true in the first place*.

## Explanatory power and explanatory goodness

### Measures of explanatory power

One way to approach the questions ‘how good is an explanation?’ and the related question ‘when is one explanation better than another?’ would be to appeal to various probabilistic measures of explanatory power. At the outset, it is worth noting that these measures are not intended to define what constitutes an explanation, but only to measure the explanatory power of a hypothesis that has been determined on other grounds to provide an explanation. With that in mind, considering an explanandum *e* and an explanans or explanatory hypothesis *h*, these measures quantify—in a sense to be discussed—the extent to which *h* explains *e*. For example, after identifying seven adequacy conditions to quantify explanatory power, Schupbach and Sprenger (2011) show that the only measure satisfying their conditions is:

1 $$\begin{aligned} {\mathcal {E}}_{1}(e,h) = \frac{P(h\vert e)-P(h\vert \lnot e)}{P(h\vert e)+P(h\vert \lnot e)}. \end{aligned}$$

Here and elsewhere, *P* represents a probability function, which is assumed to be regular (for any contingent proposition *q*, $0<P(q)<1$), *e* represents the explanandum and *h* a hypothesis that provides at least a potential explanation of *e*. Probabilities are taken to represent degrees of belief relevant to background knowledge, which is omitted in the notation for convenience. Hence, the current approach should be thought of in Bayesian terms.

Crupi and Tentori (2012) present an axiomatic representation for measures ordinally equivalent to ${\mathcal {E}}_{1}$ and then, after offering criticisms of some aspects of Schupbach and Sprenger’s approach, they present an alternative axiomatization for measures ordinally equivalent to their preferred measure:

2 $$\begin{aligned} {\mathcal {E}}_{2}(e,h) = \left\{ \begin{array}{ll} \frac{P(e\vert h)-P(e)}{1-P(e)} &{}\quad \text{ if } P(e\vert h) \ge P(e)\\ \frac{P(e\vert h)-P(e)}{P(e)} &{}\quad \text{ if } P(e\vert h) < P(e).\\ \end{array} \right. \end{aligned}$$

Cohen (2016) draws attention to another measure that had been proposed by Good (1960), who provided an axiomitzation for it, and also discussed by McGrew (2003):

3 $$\begin{aligned} {\mathcal {E}}_{3}(e,h) = \text{ log } \left[ \frac{P(e\vert h)}{P(e)} \right] . \end{aligned}$$

Cohen shows how measures ordinally equivalent to ${\mathcal {E}}_{3}$ could also be given a much simpler axiomatic representation by drawing on a result from Crupi et al. (2013).

Another measure of explanatory power was proposed by Popper (1959) as follows:

4 $$\begin{aligned} {\mathcal {E}}_{4}(e,h) = \frac{P(e\vert h) - P(e)}{P(e\vert h) + P(e)}, \end{aligned}$$

though he also considered ${\mathcal {E}}_3$ to provide an adequate definition of explanatory power as well. In fact, ${\mathcal {E}}_4$ is ordinally equivalent to ${\mathcal {E}}_3$ so both of these measures produce the same comparative explanatory judgments.

What exactly are these measures intended to quantify? According to Schupbach and Sprenger, the conception of explanatory power they have in mind is that of a ‘hypothesis’s ability to decrease the degree to which we find the explanandum surprising’ (2011, p. 108) and similarly Crupi and Tentori claim that their account captures ‘how the background surprisingness/expectedness of explanandum *e* is reduced by assuming candidate explanans *h*’ (2012, p. 375). Plausibly all these measures can be understood as attempts to capture explanatory power in the sense of ‘*h* reducing surprise in *e*’ or perhaps ‘*h* increasing expectedness of *e*’ (though see Sect. 4.3). How surprising *e* is will differ from one case to another, but the key factor is the *reduction* of surprise. We can think of this by comparing the $P(e\vert h)$, the probability of *e* given *h*, with *P*(*e*), the probability of *e* given only background knowledge. A low value of *P*(*e*) would represent a case where *e* is surprising and the lower the value of *P*(*e*) the greater the extent to which *e* is surprising. If $P(e\vert h)$ is greater than *P*(*e*), this would represent the situation where *h* reduces the surprise in *e* and the greater $P(e\vert h)$ the greater the reduction. Hence, if one is comparing two hypotheses for a given explanandum, it is the one that increases its probability most that has greater explanatory power (see Sect. 2.3). More importantly, what these measures have in common is that they attempt to quantify how well *h* would explain *e* (in the sense just noted) *if* *h* *were true*. That is, they are intended to capture something about the relationship between *h* and *e* under the assumption that *h* is true.^1^ In Good’s (1968) terminology, they are all measures of *weak* explanatory power. I will return to his distinction between weak and strong explanatory power in Sect. 3.

Before considering the suitability of these measures, it is worth commenting on some concerns about the general approach. If measures of explanatory power are concerned with reduction of surprise in the sense noted above, the problem of old evidence that is posed for Bayesianism is relevant (Glymour, 1980). Essentially, the problem is that if *e* is old evidence it is included in background knowledge so that $P(e) = 1$, which also means that $P(e\vert h) = 1$ and hence $P(h\vert e) = P(h)$, so that *e* cannot confirm *h*. Some Bayesians have appealed to variants of Garber’s (1983) approach, which sought to show that what confirms *h* is not *e* but the discovery that *h* entails *e*. However, this strategy does not help in the current context since if it is accepted that $P(e\vert h) = P(e) = 1$, then there can be no reduction in surprise. Alternatively, in the counterfactual approach to the problem, the idea is to suppose that *e* is not known to be true, removing it from background knowledge, and then to consider the impact that learning *e* would have on *h*. This approach was defended by Howson (1991), but he later rejected it because of difficulties involved in extracting *e* from background knowledge and inconsistency with a subjective Bayesian approach. Instead, he argued that ‘a minimalist version of Objective Bayesianism does straightforwardly solve the problem’ (Howson, 2017) and based his approach on earlier work by Rosenkrantz (1983). An important aspect of this approach is that a probability less than one can be legitimately assigned to *e* in the case of old evidence. If a solution along these lines is viable or if the counterfactual approach can be defended against criticisms, this would undermine the possible concern about the general approach adopted here.^2^

Another concern is that it might reasonably be doubted whether explanation could be fully analyzed in probabilistic terms. In response, it can be noted that these measures are not intended to define what constitutes an explanation, but only to measure the explanatory power of a hypothesis that has been determined on other grounds to provide an explanation. However, further objections might relate specifically to using probability to measure explanatory goodness. For example, a number of philosophers have argued that explanation is intimately tied to understanding (see, for example, Friedman, 1974; Kitcher, 1989) and, if correct, it might seem questionable whether this could be fully analyzed probabilistically. While this can be acknowledged as a potential limitation, the proposed strategy is to explore the probabilistic approach to see how far it goes. The measures of explanatory power discussed above have had some success in this regard and the hope is to extend that success further. Arguably, the suggestion that the explanatory measures described above capture ‘reduction in surprise’ and the argument that the account proposed here does justice to a number of explanatory virtues (see Sect. 4.5) might go some way to addressing this concern.

A further concern is that in the context of probabilistic explanation some have argued that low probabilities explain just as well as high probabilities (see, Jeffrey, 1969; Salmon, 1971; Railton, 1981), a viewpoint known as egalitarianism. Yet according to all the measures discussed above, explanatory power is greater for a hypothesis that confers a higher probability on a given explanandum (see Sect. 2.3). This is consistent with ‘moderate elitism’, a view defended by Strevens (2000, 2014) which does not deny that low probability events can be explained, but maintains that conferring a high probability is better. Consider, for example, a polarizer oriented at angle $\theta$ to the vertical. According to quantum theory, a probability of $\text {cos}^2(\theta )$ that an incoming, vertically polarized photon will be transmitted. On an egalitarian view, the transmission of the photon is equally well explained irrespective of whether $\theta$ is small or large, and hence the probability of transmission large or small (though not zero) respectively. A motivation for the egalitarian view is that in the low probability scenario, there are no further relevant factors that could be cited. However, there also seems to be a clear motivation for saying that the transmission is better explained by small $\theta$ (and hence high probability). Suppose we know that $\theta$ was either small (oriented very close to the vertical) or large (very close to the horizontal) and that both hypotheses are equally plausible in light of background knowledge. The transmission of the vertically polarized photon would be surprising given large $\theta$, but much less surprising given small $\theta$. Furthermore, the reduction of surprise would be greater given small $\theta$ if multiple vertically polarized photons were all transmitted. Hence, thinking about explanation in terms of reduction of surprise (as well as in the context of IBE, see introduction) gives some reason for thinking that the small $\theta$ hypothesis provides a better explanation in this case. A detailed discussion of these issues is beyond the scope of this paper, but these points suggest that in at least some cases there is justification for pursuing the current approach.^3^

Could these measures be used to make judgments about explanatory goodness? According to Schupbach and Sprenger (2011), their goal is to propose a measure of explanatory power that ‘would clarify the conditions under which hypotheses are judged to provide strong versus weak explanations’ (p. 106). They further claim that an appropriate analysis of explanatory power ‘would also clarify the meaning of comparative explanatory judgments such as “hypothesis A provides a better explanation of this fact than does hypothesis B”’ (p. 106). However, they also point out that they ‘take no position on whether our analysis captures the notion of explanatory power generally; it is consistent with our account that there be other concepts that go by this name but which do not fit our measure’ (p. 106). According to Schupbach (private communication), their measure ${\mathcal {E}}_1$ is appropriate for making judgments about explanatory goodness in some cases, such as those where priors are not accessible or whenever agents have knowingly ungrounded subjective priors, but in other cases judgments of explanatory goodness may require other factors to be taken into account. In particular, they may require a trade-off between explanatory power in their sense, which corresponds to Good’s notion of weak explanatory power, and the improbability of the hypothesis since a hypothesis with high explanatory power might not rank so well in terms of overall explanatory goodness if it has a low prior probability. Following Good (1968), I will assume that the probability and complexity of a hypothesis are inversely related, so the more improbable a hypothesis, the greater its complexity.^4^ Achieving an appropriate trade-off between weak explanatory power and improbability/complexity is the focus of the current paper.

In the rest of this section, I will highlight the need for such a trade-off in a wide range of cases and to that end I will focus on what the four measures identified so far have in common.

### Entailment

${\mathcal {E}}_{1}$ and ${\mathcal {E}}_{2}$ are maximal in cases where *h* entails *e*, while ${\mathcal {E}}_{3}$ and ${\mathcal {E}}_4$ take on their greatest values for a given *e* in cases where *h* entails *e*. Although this is appropriate for the specific concept of explanatory power these measures attempt to explicate (reduction of surprise), it seems to be a distinct weakness if one is trying to evaluate the overall goodness of an explanation or to compare explanations with each other. We can often distinguish between how well two hypotheses explain the evidence in cases where both of them entail the evidence. For example, explanationists typically cite simplicity as an explanatory virtue that could discriminate in such cases. If a conspiracy theory is deliberately constructed in such a way that *if it were true*, it would entail the explanandum in question, it would still be reasonable to think that it is a very poor explanation if it is *very unlikely to be true* in the first place.

### Equal likelihoods and irrelevant conjunction

Closely related to the case of entailment, it turns out that all four measures satisfy the following condition for a given *e*: ${\mathcal {E}}(e,h_1) = {\mathcal {E}}(e,h_2)$ if and only if $P(e\vert h_1) = P(e\vert h_2)$. In fact, this condition is enshrined in the principle of positive relevance, which is used in the axiomatization of these measures (Cohen, 2016):

Positive relevance. ${\mathcal {E}}(e,h_1) \gtreqless {\mathcal {E}}(e,h_2)$ if and only if $P(e\vert h_1) \gtreqless P(e\vert h_2)$.

An application of *positive relevance* gives rise to another important feature of all four measures known as *irrelevant conjunction*. It says that conjoining an irrelevant hypothesis, $h_2$, to a given hypothesis, $h_1$, has no effect on $h_1$’s (weak) explanatory power:^5^

Irrelevant conjunction. If $h_2$ is probabilistically independent of *e*, $h_1$ and their conjunction, then ${\mathcal {E}}(e,h_1 \wedge h_2) = {\mathcal {E}}(e,h_1)$.

It is easy to see that this follows from positive relevance since $P(e\vert h_1\wedge h_2) = P(e\vert h_1)$ when $h_2$ is probabilistically independent of *e* and $h_1$, and hence given positive relevance that ${\mathcal {E}}(e,h_1\wedge h_2) = {\mathcal {E}}(e,h_1)$. Schupbach and Sprenger argue for this condition on the grounds that ‘$h_1 \wedge h_2$ will not make *e* any more or less surprising than $h_1$ by itself already does’ (2011, p. 110) and hence has no effect on explanatory power. Crupi and Tentori agree, noting that ‘it does not alter the degree to which *e* is explained’ (2012, p. 367). While this is appropriate for measures of explanatory power that seek to explicate how well a hypothesis would explain the explanandum *if the hypothesis were true*, it seems clear that adding an irrelevant hypothesis results in a worse explanation overall. Why? Because once again considerations of simplicity and plausibility come into play. Let *e* be a description of the bending of light from a distant source by the sun and $h_1$ an explanation of this by Einstein’s theory of general relativity. Let $h_2$ be the hypothesis that I have an identical twin elsewhere in the universe. All four measures judge that Einstein’s theory explains the bending of light to the same extent that the conjunction of Einstein’s theory and the hypothesis about my identical twin explains it. A plausible measure of explanatory goodness should show that this conjunction provides a worse explanation.

The foregoing discussion suggests a satisfactory measure of explanatory goodness should capture the idea that in the case of irrelevant conjunction the more concise explanation is better:

Concise explanation. If $h_2$ is probabilistically independent of *e*, $h_1$ and their conjunction, then ${\mathcal {E}}(e,h_1 \wedge h_2) \le {\mathcal {E}}(e,h_1)$, with equality only in the case where $P(h_2\vert h_1) = 1$.

However, the more general point that applies to all cases where two hypotheses have equal likelihoods is that explanatory factors such as simplicity or plausibility can discriminate between them. This motivates the following adequacy condition for a measure of explanatory goodness based on the relevance of the initial plausibility of the hypotheses as measured by their prior probabilities given only background knowledge:

Initial plausibility. If $P(e\vert h_1) = P(e\vert h_2)$, then ${\mathcal {E}}(e,h_1) \gtreqless {\mathcal {E}}(e,h_2)$ if and only if $P(h_1) \gtreqless P(h_2)$.

Note that the *concise explanation* condition follows from *initial plausibility*. I will explore the role of prior probabilities further below.

### Probabilistic relevance

Could a hypothesis which is negatively relevant to *e* provide a better explanation than one which is positively relevant to *e*? Consider a bag consisting of 99 fair coins and one coin with a bias towards heads such that its objective chance of landing heads is 0.51. A coin is selected at random and, on being tossed, lands heads. Consider the hypotheses: ‘the selected coin is fair’ ($h_1$) and ‘the selected coin is biased’ ($h_2$). Note that $h_1$ is negatively related to the observation since $P(e\vert h_1) = 0.5 < 0.5001 = P(e)$ while $h_2$ is positively related to it. Thinking of explanation in terms of how well the hypotheses would account for the explanandum if they were true, which is what the four measures specified earlier seem to explicate, $h_2$ provides the better explanation. However, this does not take into account the prior improbability of $h_2$, which is relevant if we are assessing the overall goodness of the explanations. In this sense, given the very small difference in the likelihoods and the much greater prior probability of $h_1$, it is plausible to think that $h_1$ provides a much better explanation overall. Arguably, a trade-off needs to be made between probabilistic relevance and complexity (in the sense of lower probability) when evaluating an explanation, though how exactly that trade-off should be made is not immediately obvious. I explore this matter in Sects. 2.5 and 3.^6^

Even though $h_1$ seems to provide a better overall explanation than $h_2$, there is also something deficient about $h_1$ as an explanation due to its negative relevance to the explanandum and this should feature in any plausible account of explanatory goodness. I will return to this point in Sect. 4.4.

### Striking the balance

While it is perfectly reasonable to consider explanatory power in the sense explicated by measures ${\mathcal {E}}_1 - {\mathcal {E}}_4$ (weak explanatory power) as a factor in explanatory goodness, the focus in this section has been on the need for a trade-off between weak explanatory power and complexity. Or to put it another way, a measure of explanatory goodness should combine weak explanatory power and prior probability in an appropriate manner. The *initial plausibility* condition specifies how the priors can play a role when the likelihoods are equal, but how should they be taken into account more generally?

It might be thought that Bayes’ theorem provides an answer since it essentially combines ${\mathcal {E}}_3$ with the prior probability. In that case, the goodness of an explanation would be identified with its posterior probability. But there are good reasons to reject this approach. First, while priors are relevant to explanatory goodness, arguably this approach gives too much weight to priors via Bayes’ theorem. While explanationists would like to think that the best explanation would often turn out to be the most probable hypothesis, it certainly seems possible that in at least some scenarios this might fail to be the case. Second, it also seems that in some cases a conjunctive explanation that combines two compatible hypotheses, $h_1 \wedge h_2$ say, could turn out to be a better explanation than either $h_1$ or $h_2$, yet this is ruled out if explanatory goodness is identified with posterior probability.

One way of putting this is as follows. If $h_1$ and $h_2$ have equal posteriors then since $P(e\vert h_1)\cdot P(h_1) = P(e\vert h_2)\cdot P(h_2)$, if we were to treat them as equal in terms of explanatory goodness, we would essentially be giving the priors as much importance as likelihoods. While I have argued that excluding priors is too extreme in one direction, giving them this much of a role is arguably too extreme in the other direction; a better balance is needed. In light of these considerations, it seems reasonable to use likelihoods to discriminate between hypotheses with equal posterior probabilities. Hence, despite my concerns about the *positive relevance* condition, it does seem appropriate to apply it in cases where the priors or the posteriors of the hypotheses are equal. This suggests the following restricted version of the positive relevance condition:

Restricted positive relevance. If $P(h_1) = P(h_2)$ or $P(h_1\vert e) = P(h_2\vert e)$, then ${\mathcal {E}}(e,h_1) \gtreqless {\mathcal {E}}(e,h_2)$ if and only if $P(e\vert h_1) \gtreqless P(e\vert h_2)$.

Now we are in a position to consider how to make the appropriate trade-off between weak explanatory power and improbability/complexity.

## A Good approach to good explanation

The mathematician and World War II cryptologist I. J. Good made significant contributions to this topic. I have already drawn attention to his measure in Eq. (3), which is probably the best known measure of explanatory power. Based on the desiderata he set out in his 1960 paper, he argued that this measure was ‘essentially the only possible explicatum for explanatory power’ (Good, 1960, p. 320). However, in another paper in 1968 he distinguished between explanatory power in the weak sense (weak explanatory power) and the strong sense (strong explanatory power) and noted that ‘the double meaning of “explanatory power” has previously been overlooked’ (Good, 1968, p. 124). By weak explanatory power, he meant that the explanatory power of a hypothesis *h* is ‘unaffected by cluttering up [*h*] with irrelevancies’, while strong explanatory power ‘*is* affected by the cluttering’ (Good, 1968, p. 123).

When is a hypothesis ‘cluttered up with irrelevancies’? One of Good’s desiderata (axiom 10 in the 1968 paper) provides the answer and hence the key distinction between weak and strong measures of explanatory power. This desideratum is essentially the *irrelevant conjunction* condition specified earlier. So *irrelevant conjunction* must be satisfied by a weak measure of explanatory power since it is unaffected by the inclusion of an irrelevant hypothesis (clutter). However, strong measures do not satisfy *irrelevant conjunction*, but instead take the prior probability into account to penalize the inclusion of an irrelevant hypothesis.

Note that strong explanatory power is intended to penalize not just the addition of irrelevant hypotheses, but also improbable/complex hypotheses more generally. An analogy with model selection might help to motivate this approach. By adopting a sufficiently complex model, it is possible to obtain an excellent fit to the data, but in doing so one is likely to over-fit the model to noise in the data. Hence, a trade-off between how well the model fits the data and the complexity of the model is sought and this can be achieved by penalizing models for their complexity. In Bayesian model selection, more complex models can be assigned lower probabilities so they are penalized more. This trade-off is closely related to that needed here. For example, in many cases it is possible to come up with an ad hoc hypothesis or conspiracy theory that has been deliberately constructed to entail the explanandum (see Sect. 2.2) even though the hypothesis itself is very improbable. To avoid this, hypotheses need to be penalized for their improbability/complexity. In model selection, this is often expressed in terms of Ockham’s razor and as we will see this is also how Good refers to his approach.

The strong measure advocated by Good is:

5 $$\begin{aligned} {\mathcal {E}}_{5}(e,h) = \text{ log } \left[ \frac{P(e\vert h)\cdot P(h)^\gamma }{P(e)} \right] , \end{aligned}$$

where $0< \gamma < 1$ is a constant and so (5) provides a continuum of measures of strong explanatory power. According to Good, ‘the constant $\gamma$ measures the degree to which the simplicity of the hypothesis is regarded as desirable ... as compared with its weak explanatory power’ (Good, 1968, p. 130). If $\gamma = 0$ were permitted then ${\mathcal {E}}_5$ would just be Good’s weak measure, ${\mathcal {E}}_3$, so weak explanatory power can be seen as a limiting case of strong explanatory power. Furthermore, requiring $\gamma > 0$ means that ${\mathcal {E}}_5$ satisfies the *concise explanation* condition. Also, if $\gamma = 1$ were permitted then ${\mathcal {E}}_5$ would just be the log of posterior probability and so requiring $\gamma < 1$ means that ${\mathcal {E}}_5$ satisfies the *restricted positive relevance* condition.

Relating this to the discussion in Sect. 2, we can see that the *positive relevance* condition is closely related to weak explanatory power since it entails the *irrelevant conjunction* condition. By contrast, the *initial plausibility* condition is closely related to strong explanatory power since it entails the *concise explanation* condition. And while a strong measure should not satisfy the *positive relevance* condition, it should nevertheless satisfy the *restricted positive relevance* condition.

The four measures discussed in Sect. 2 are appropriate if one is making judgments about weak explanatory power and so debates about their relative merits are to be understood in that light. However, if instead one is interested in when one hypothesis provides a better overall explanation of a given explanandum than another hypothesis does, then it seems that something along the lines of Good’s strong sense of explanatory power is needed. In fact, Good proposes what he calls a ‘sharpened version of “Ockham’s razor” which is that if our primary purpose is explanation we should select the hypothesis (among those we know) which has the maximum strong explanatory power’ (1968, p. 123).

A measure motivated by considerations of coherence provides another example of a measure of strong explanatory power (Glass, 2021):

6 $$\begin{aligned} {\mathcal {E}}_{6}(e,h) = P(e\vert h) \cdot P(h\vert e) = \frac{P(e\vert h)^2 \cdot P(h)}{P(e)}. \end{aligned}$$

Strictly speaking, ${\mathcal {E}}_6$ was not proposed as a measure of explanatory power as such, but rather as a measure for ranking hypotheses as explanations of an explanandum *e*. It is easy to show that ${\mathcal {E}}_6$ satisfies the *initial plausibility*, *concise explanation* and *restricted positive relevance* conditions. Furthermore, when comparing $h_1$ and $h_2$ as explanations of *e* it judges $h_1$ to be better than $h_2$ if and only if:

7 $$\begin{aligned}&P(e\vert h_1)^2 \cdot P(h_1)> P(e\vert h_2)^2 \cdot P(h_2) \nonumber \\ \Leftrightarrow \;\;&P(e\vert h_1) \cdot P(h_1)^{1/2} > P(e\vert h_2) \cdot P(h_2)^{1/2}, \end{aligned}$$

and hence it provides the same ordering of explanations as Good’s strong measure, ${\mathcal {E}}_5$, if we set $\gamma = \nicefrac {1}{2}$, which Good describes as the simplest explicatum. I will return to this point in Sect. 4.4.

The overlap coherence measure was also proposed for ranking explanations. For a hypothesis *h* and explanandum *e* the overlap coherence is given by (Glass, 2002; Olsson, 2002):

8 $$\begin{aligned} {\mathcal {E}}_7(e,h) = \frac{P(h\wedge e)}{P(h \vee e)}. \end{aligned}$$

Like ${\mathcal {E}}_5$ and ${\mathcal {E}}_6$, it also satisfies the *initial plausibility*, *concise explanation* and *restricted positive relevance* conditions and so can be considered as another strong measure of explanatory power. So Good’s strong measure is not the only measure of strong explanatory power and hence further reasons need to be given in its defence. I now turn to that task.

## A defence of Good’s strong measure

### Deriving Good’s strong measure

In his 1968 paper, Good adopted a two stage strategy to show that a measure of strong explanatory power must be a monotonically increasing function of his ${\mathcal {E}}_5$ measure. First, he drew on his 1960 paper where he showed that a weak measure of explanatory power must be a monotonically increasing function of his ${\mathcal {E}}_3$ measure based on ten axioms or desiderata for such a measure. Then he made some assumptions about a strong measure and its relation to his weak measure in order to derive his result concerning ${\mathcal {E}}_5$.

Here I want to present two new derivations that relate more closely to some of the desiderata for measures of explanatory power found in the recent literature. The first approach does not require establishing ${\mathcal {E}}_3$ as a measure of weak explanatory power, but rather a property of it, which is sufficient to establish ${\mathcal {E}}_5$. The second follows Good’s strategy of first establishing ${\mathcal {E}}_3$, in this case drawing on a result by Crupi et al. (2013), before using Good’s result to establish ${\mathcal {E}}_5$ as a measure of strong explanatory power.

The first condition is based on Crupi and Tentori (2012). It is a formal assumption about measures of weak and strong explanatory power, which I will denote as ${\mathcal {E}}_W$ and ${\mathcal {E}}_S$ respectively.

(A1) Let *L* be a propositional language and $L_c$ the contingent formulas in *L*. Let ***P*** be the set of regular probability functions that can be defined over *L* and let ${\mathcal {E}}_W: {L_c \times L_c \times {{\varvec{P}}}} \rightarrow {\mathbb {R}}$ and ${\mathcal {E}}_S: {L_c \times L_c \times {{\varvec{P}}}} \rightarrow {\mathbb {R}}$. There exist continuous, differentiable functions *w* and *s* such that, for any $e, h \in L_c$ and any $P \in {{\varvec{P}}}$, ${\mathcal {E}}_W(e,h) = w[P(e \wedge h), P(h), P(e)]$ and ${\mathcal {E}}_S(e,h) = s[P(e \wedge h), P(h), P(e)]$.

In terms of the dependence on $P(e \wedge h)$, *P*(*h*) and *P*(*e*), A1 just says that ${\mathcal {E}}_W$ and ${\mathcal {E}}_S$ are functions of absolute and conditional probabilities of logical combinations of *h* and *e* since all of these probabilities are determined by $P(e \wedge h)$, *P*(*h*) and *P*(*e*). The requirement of continuity and differentiability enables us to take advantage of part of Good’s proof and ensures that the functions are well-behaved.

The second condition requires that ${\mathcal {E}}_S$ depend only on ${\mathcal {E}}_W$ and *P*(*h*). This is motivated by the distinction between a weak and strong measure of explanatory power since the latter should take into account the simplicity/complexity of the hypothesis in addition to its weak explanatory power.

(A2) ${\mathcal {E}}_S$ can be expressed as a function of ${\mathcal {E}}_W$ and *P*(*h*) so that ${\mathcal {E}}_S(e,h) = s[P(e \wedge h), P(h), P(e)] = s_W[{\mathcal {E}}_W(e,h),P(h)]$.

A possible objection to this condition is that while it might be accepted that ${\mathcal {E}}_S$ should depend on ${\mathcal {E}}_W$ and *P*(*h*), it might be questioned whether it should only depend on these two factors. However, we need to distinguish conceptually between a measure of strong explanatory power and a measure of overall explanatory goodness. Given Good’s account of strong explanatory power, this condition seems unobjectionable. Whether a strong measure will turn out to provide a plausible measure of overall explanatory goodness will depend on how well it captures various explanatory virtues (see Sect. 4.5).

The third condition says that a weak measure of explanatory power should treat probabilistic independence between *e* and *h* as a special case by assigning it a fixed, neutral value. This clearly holds for ${\mathcal {E}}_1 - {\mathcal {E}}_4$ since they are measures of probabilistic relevance.

(A3) ${\mathcal {E}}_W$ has a fixed, neutral point $\alpha$ such that ${\mathcal {E}}_W(e,h) = \alpha$ if and only if *h* and *e* are probabilistically independent.

Suppose that $h_1$ provides an explanation of $e_1$ and $h_2$ provides an explanation of $e_2$, but that $h_2$ and $e_2$ are irrelevant to $h_1$ and $e_1$. The fourth condition says that the degree to which $h_1 \wedge h_2$ explains $e_1 \wedge e_2$ is a function of the degree to which $h_1$ explains $e_1$ and the degree to which $h_2$ explains $e_2$ and that this applies for both weak and strong measures of explanatory power. Such a condition is discussed by Good (1968) in the context of strong explanatory power and by Cohen (2016), who presents a generalized version of this condition for an arbitrary number of explanandum-explanans pairs. Formally, it can be stated as follows:

(A4) If $h_2$ and $e_2$ are each probabilistically independent of $h_1$, $e_1$ and their conjunction, then ${\mathcal {E}}_W(e_1 \wedge e_2, h_1 \wedge h_2)$ can be expressed as a function, $w_c$, of ${\mathcal {E}}_W(e_1, h_1)$ and ${\mathcal {E}}_W(e_2, h_2)$ so that ${\mathcal {E}}_W(e_1 \wedge e_2, h_1 \wedge h_2) = w_c[{\mathcal {E}}_W(e_1, h_1),{\mathcal {E}}_W(e_2, h_2)]$, where $w_c$ is strictly increasing in each argument when the other argument is fixed and non-extreme (i.e. neither its maximum or minimum value) and non-decreasing otherwise. Similarly there is a corresponding function, $s_c$, for ${\mathcal {E}}_S$ so that ${\mathcal {E}}_S(e_1 \wedge e_2, h_1 \wedge h_2) = s_c[{\mathcal {E}}_S(e_1, h_1),{\mathcal {E}}_S(e_2, h_2)]$.

In some cases, it seems very appropriate to combine independent explanations in this way. Cohen (2016), for example, highlights its relevance to sets of experiments where each is carried out in a different laboratory and has a separate hypothesis. However, Cohen does not propose this property as a necessary requirement for measures of explanatory power since he sees its virtue as being one of convenience. Certainly, it can be very convenient if a measure decomposes into products or sums, as is the case for Good’s measures. For Good’s weak measure we have:

9 $$\begin{aligned} {\mathcal {E}}_3(e_1 \wedge e_2, h_1 \wedge h_2)= & {} \text{ log } \left[ \frac{P(e_1 \wedge e_2\vert h_1 \wedge h_2)}{P(e_1 \wedge e_2)} \right] \nonumber \\= & {} \text{ log } \left[ \frac{P(e_1\vert h_1)}{P(e_1)} \right] + \text{ log } \left[ \frac{P(e_2\vert h_2)}{P(e_2)} \right] \nonumber \\= & {} {\mathcal {E}}_3(e_1, h_1) + {\mathcal {E}}_3(e_2, h_2), \end{aligned}$$

when the appropriate independence relationships hold and it is easy to show that the corresponding result holds for his strong measure as well. While such a decomposition is convenient, there are good reasons to think that A4 should indeed be a necessary condition for measures of explanatory power.

Suppose a patient reports two symptoms, $e_1$ and $e_2$. Whatever the patient might think, suppose the doctor has good reason to believe that there is no dependence between these symptoms and is able to explain them by conditions $h_1$ and $h_2$ respectively, which again are independent of each other and of the evidence they do not explain. In such a case, it is reasonable to combine these independent hypotheses to explain the symptoms to the patient. Furthermore, how well they explain the symptoms is very plausibly taken to be an increasing function of each explanation. For example, suppose the doctor had two potential explanations, $h_2$ and $h_3$, for $e_2$ and that both satisfied the relevant independence conditions with $h_1$ and $e_1$. It seems clear that if $h_2$ provides a better explanation of $e_2$ than $h_3$ does, then the combined explanatory power of $h_1$ and $h_2$ would be greater than that of $h_1$ and $h_3$.

So the importance of A4 lies not merely its convenience, but rather in the plausibility of requiring that when explanations are combined and the relevant independence conditions are met, explanatory power should be an increasing function of each explanation. This becomes clear when we see scenarios where measures such as ${\mathcal {E}}_1$ and ${\mathcal {E}}_2$ violate A4.

#### Example 1

Suppose that $(e_1,h_1)$, $(e_2,h_2)$ and $(e_2',h_2')$ are three explanandum-explanans pairs satisfying the relevant independence conditions for A4. These can be thought of as three pairs of symptoms and corresponding conditions that explain them, with each of the three pairs being irrelevant to the other pairs. Suppose $P(e_1\vert h_1) = 1$, $P(e_1) = 0.5$, $P(e_2\vert h_2) = 0.4$, $P(e_2) = 0.2$, $P(e_2'\vert h_2') = 0.8$ and $P(e_2') = 0.75$. Clearly, ${\mathcal {E}}_1(e_1,h_1) = {\mathcal {E}}_2(e_1,h_1) = 1$. Also, ${\mathcal {E}}_1(e_2,h_2) \approx 0.455 > 0.143 \approx {\mathcal {E}}_1(e_2',h_2')$. Combining explanations, we find that ${\mathcal {E}}_1(e_1 \wedge e_2,h_1 \wedge h_2) \approx 0.714 < 0.739 \approx {\mathcal {E}}_1(e_1 \wedge e_2',h_1 \wedge h_2')$. Hence, since ${\mathcal {E}}_1(e_2,h_2) > {\mathcal {E}}_1(e_2',h_2')$, but ${\mathcal {E}}_1(e_1 \wedge e_2,h_1 \wedge h_2) < {\mathcal {E}}_1(e_1 \wedge e_2',h_1 \wedge h_2')$, ${\mathcal {E}}_1$ violates A4. The same is true of ${\mathcal {E}}_2$ since ${\mathcal {E}}_2(e_2,h_2) = 0.25 > 0.2 = {\mathcal {E}}_2(e_2',h_2')$, while ${\mathcal {E}}_2(e_1 \wedge e_2,h_1 \wedge h_2) \approx 0.333 < 0.68 = {\mathcal {E}}_2(e_1 \wedge e_2',h_1 \wedge h_2')$.

So although (a) $h_1$ explains $e_1$ and (b) $h_2$ explains $e_2$ better than $h_2'$ explains $e_2'$, ${\mathcal {E}}_1$ and ${\mathcal {E}}_2$ counterintuitively give the result that (c) $h_1 \wedge h_2$ explains $e_1 \wedge e_2$ less well than $h_1 \wedge h_2'$ explains $e_1 \wedge e_2'$. In fact, matters are worse than this since, according to ${\mathcal {E}}_1$ and ${\mathcal {E}}_2$, the combination of two poorer explanations can be better than the combination of two better explanations. These results provide good reasons for adopting A4 as a necessary requirement for both weak and strong measures of explanatory power.

In the discussion so far, I have argued for A4 by appealing to examples that are intended to highlight its plausibility. However, since ${\mathcal {E}}_1$ and ${\mathcal {E}}_2$ violate A4, these examples provide counterexamples to these measures. A possible response is to say that even in terms of weak explanatory power ${\mathcal {E}}_1$ and ${\mathcal {E}}_2$ are better thought of as explications of a different concept from the one being proposed here and hence from ${\mathcal {E}}_3$. I think this is a reasonable response and will return to it in Sect. 4.2 where I discuss the fact that A4 leads to a property of ${\mathcal {E}}_3$ and ${\mathcal {E}}_5$ that has been criticized in the literature.

The final two conditions were discussed earlier:

(A5) ${\mathcal {E}}_S$ satisfies initial plausibility (see Sect. 2.3).

(A6) ${\mathcal {E}}_S$ satisfies restricted positive relevance (see Sect. 2.4).

Based on these assumptions and recalling that ${\mathcal {E}}_S$ is a function of ${\mathcal {E}}_W$ and *P*(*h*) as expressed in A2, we then get the following theorem for Good’s strong measure of explanatory power, ${\mathcal {E}}_5$.^7^

#### Theorem 1

If ${\mathcal {E}}_W$ and ${\mathcal {E}}_S$ are weak and strong measures of explanatory power respectively that satisfy A1 - A6, then ${\mathcal {E}}_S$ is a monotonically increasing function of Good’s strong measure, ${\mathcal {E}}_5$.^8^

For an alternative way to derive Good’s measure, consider the following conditions for a weak measure of explanatory power:

(A7) For any $e, h_1, h_2 \in L_c$ and $P \in {{\varvec{P}}}$, ${\mathcal {E}}_W(e,h_1) \gtreqless {\mathcal {E}}_W(e,h_2)$ if and only if $P(e\vert h_1) \gtreqless P(e\vert h_2)$, i.e. ${\mathcal {E}}_W$ satisfies positive relevance (see Sect. 2.3).

(A8) For any $e_1, e_2, h \in L_c$ and $P \in {{\varvec{P}}}$, ${\mathcal {E}}_W(e_1,h) \gtreqless {\mathcal {E}}_W(e_2,h)$ if and only if $P(h\vert e_1) \gtreqless P(h\vert e_2)$.

A7 (positive relevance) seems like a very plausible condition for weak explanatory power. Of course, positive relevance was criticized in Sect. 2.3 in the context of overall explanatory goodness, but it is appropriate to retain it as a condition for weak explanatory power. Furthermore, all of the weak measures considered in this paper, ${\mathcal {E}}_1 - {\mathcal {E}}_4$, satisfy A7.

What about A8? In some particular cases, there is clear justification for A8 if we think of weak explanatory power in terms of reducing surprise. If $P(e_1) = P(e_2)$, then for ${\mathcal {E}}_W(e_1,h) > {\mathcal {E}}_W(e_2,h)$ seems to require that $P(e_1\vert h) > P(e_2\vert h)$, from which it follows that $P(h\vert e_1) > P(h\vert e_2)$. Similarly, if $P(e_1\vert h) = P(e_2\vert h)$, then ${\mathcal {E}}_W(e_1,h) > {\mathcal {E}}_W(e_2,h)$ seems to require that $P(e_1) < P(e_2)$, from which it again follows that $P(h\vert e_1) > P(h\vert e_2)$. More generally, however, A8 is widely accepted as a necessary requirement for measures of the degree to which *e* confirms *h*. Indeed, so central is it that Crupi and Tentori (2014) include it as part of their definition of confirmation, calling it *final probability*. In the present context, A8 can then be understood as stating a fundamental relationship between explanation and confirmation. It ensures that if *h* provides explanations of $e_1$ and $e_2$, then it weakly explains (reduces the surprise of) $e_1$ better than $e_2$ exactly when $e_1$ provides greater confirmation of *h* than does $e_2$. This seems very plausible indeed since it is precisely the ability to explain otherwise very surprising phenomena that can provide strong confirmation of a hypothesis.^9^

Using A7 and A8 we then get the following theorem for Good’s strong measure of explanatory power, ${\mathcal {E}}_5$.

#### Theorem 2

If ${\mathcal {E}}_W$ and ${\mathcal {E}}_S$ are weak and strong measures of explanatory power respectively that satisfy A1, A2, A4 - A8 then ${\mathcal {E}}_W$ is a monotonically increasing function of Good’s weak measure, ${\mathcal {E}}_3$, and ${\mathcal {E}}_S$ is a monotonically increasing function of Good’s strong measure, ${\mathcal {E}}_5$.^10^

### Irrelevant evidence

The most significant objection to Good’s weak measure of explanatory power, but which applies equally to his strong measure, is the problem of irrelevant evidence due to Schupbach and Sprenger (2011). Let *e* be a general description of Brownian motion and *h* be Einstein’s atomic explanation of it. Assuming $P(e\vert h)/P(e) \gg 1$, Good’s weak measure correctly judges this to be a good explanation. However, let $e'$ be the irrelevant proposition that the mating season for an American green tree frog takes place from mid-April to mid-August. According to Good’s measures (weak and strong) this has no bearing on the explanatory power of Einstein’s account since $P(e \wedge e'\vert h) / P(e \wedge e') = P(e\vert h)/P(e)$. By contrast, according to the measures ${\mathcal {E}}_1$ and ${\mathcal {E}}_2$, the addition of $e'$ reduces the explanatory power.

Is this consequence of Good’s measures as counterintuitive as Schupbach and Sprenger claim? I will respond by trying to show that there is a very plausible way to make sense of the alleged counterexample. Unlike the measures ${\mathcal {E}}_1$ and ${\mathcal {E}}_2$, Good’s measures place no upper boundary on the degree of explanatory power. If there are two explananda, $e_1$ and $e_2$, Good’s weak measure can be expressed as

10 $$\begin{aligned} {\mathcal {E}}_{3}(e_1 \wedge e_2,h)&= \text{ log } \left[ \frac{P(e_1 \wedge e_2\vert h)}{P(e_1 \wedge e_2)} \right] = \text{ log } \left[ \frac{P(e_2\vert h, e_1)}{P(e_2\vert e_1)} \right] + \text{ log } \left[ \frac{P(e_1\vert h)}{P(e_1)} \right] ,\nonumber \\&= {\mathcal {E}}_3(e_2,h\vert e_1) + {\mathcal {E}}_3(e_1,h), \end{aligned}$$

where ${\mathcal {E}}_3(e_2,h\vert e_1)$ represents the conditional weak explanatory power, i.e. the degree to which *h* weakly explains $e_2$ after conditioning on $e_1$. Hence, the weak explanatory power of *h* for $e_1 \wedge e_2$ is obtained by adding the degree to which it weakly explains $e_2$ conditional on $e_1$ to the degree to which it weakly explains $e_1$. Good (1960) refers to this as strict additivity of the first kind. Clearly, there is always scope for the explanatory power to be greater when $e_2$ is included than it was in the case of just $e_1$. If the degree to which *h* explains $e_2$ given $e_1$ is positive, then the explanatory power increases, if it is negative, explanatory power decreases, and if it is zero, explanatory power remains unchanged. Even if *h* entails $e_1$, the explanatory power could increase further. For example, if *h* also entailed $e_2$ then it would increase further (provided $e_1$ did not entail $e_2$).

Returning to the earlier example, while Einstein’s atomic account, *h*, provides an excellent explanation of *e*, which gives a general description of Brownian motion, its explanatory power would be increased further if it could explain additional relevant evidence. For example, Einstein’s account explained not only the general phenomenon of Brownian motion, but also the much more specific results of Perrin’s 1908 experiments to determine the mean square displacement of particles undergoing Brownian motion and its relation to Avogadro’s number, which further confirmed the atomic theory. By contrast, the explanatory power of Einstein’s account would have decreased had there been additional evidence, $e_2$ say, for which Einstein’s account had negative explanatory power (given *e*). So conjoining the original evidence *e* with additional positively relevant evidence explained by *h* would increase the explanatory power of *h*, while conjoining *e* with additional negatively relevant evidence such as $e_2$ would decrease the explanatory power of *h*. What effect should conjoining *e* with a proposition about American green tree frogs, which is completely irrelevant to *h*, have on the explanatory power of *h*? According to ${\mathcal {E}}_3$, it has no effect whatsoever, which seems very reasonable.

However, measures ${\mathcal {E}}_1$ and ${\mathcal {E}}_2$ are not additive and so give very different results. In fact, this gives rise to a counterintuitive feature of these measures relating to entailment. If a hypothesis, *h* entails evidence, *e*, then conjoining *e* with further evidence cannot increase the explanatory power of *h*, no matter how well *h* explains this further evidence. And so if Einstein’s account entails a general description of Brownian motion, then its explanatory power would not be increased by conjoining this evidence with Perrin’s findings relating to Avogadro’s number.

Furthermore, suppose a hypothesis entails an explanandum that is not at all surprising because it has a high prior probability in light of background knowledge, then its explanatory power cannot be enhanced by entailing a further surprising explanandum. Planetary orbits ($e_1$) that could be derived from Newton’s theory could also be derived from Einstein’s theory (*h*) and so the explanatory power of Einstein’s theory would be one according to ${\mathcal {E}}_1$ and ${\mathcal {E}}_2$. However, the perihelion of Mercury ($e_2$) could also be derived from Einstein’s theory, but according to ${\mathcal {E}}_1$ and ${\mathcal {E}}_2$ its explanatory power for $e_1 \wedge e_2$ would not be any greater than it was for $e_1$. We might call this the problem of *relevant evidence*.

In summary, there is a very plausible way to make sense of the irrelevant evidence issue from the perspective of Good’s weak measure (and hence his strong measure too). Furthermore, I have argued that the non-additive nature of measures such as ${\mathcal {E}}_1$ and ${\mathcal {E}}_2$ can give rise to counterintuitive judgments about explanatory power and, in particular, the problem of relevant evidence. However, maybe there is another way to view these differences. Although ${\mathcal {E}}_1$, ${\mathcal {E}}_2$ and Good’s measure, ${\mathcal {E}}_3$, as well as ${\mathcal {E}}_4$, are all weak measures of explanatory power, they may nevertheless be explicating different concepts. Arguably, measures such as ${\mathcal {E}}_1$ and ${\mathcal {E}}_2$ are better understood as explications of the degree to which *h* entails *e*.^11^ However, if one wants a weak measure of explanatory power that does justice to explanatory scope and so increases appropriately as it explains more evidence, then an additive measure such as Good’s weak measure, ${\mathcal {E}}_3$, is suitable since it satisfies Eq. (10) as well as A4. I will also argue below that ${\mathcal {E}}_3$ has advantages in terms of explicating reduction of surprise.^12^

### Explanatory power and information

Good (1968) considers how his measures of weak and strong explanatory relate to semantic information. According to one very widely used account, the semantic information or information content of *h* is given by Bar-Hillel and Carnap (1953):

11 $$\begin{aligned} \text{ Inf }\;(h) = - \text{ log } P(h) \end{aligned}$$

for a probability distribution *P*, while the information content of *h* given *e* is:

12 $$\begin{aligned} \text{ Inf }\;(h\vert e) = - \text{ log } P(h\vert e). \end{aligned}$$

The information concerning *h* provided by *e* is given by:

13 $$\begin{aligned} \text{ Inf }\;(h,e) = \text{ log } \left[ \frac{P(e\vert h)}{P(e)} \right] , \end{aligned}$$

which Good also calls the mutual information between *h* and *e* since it is symmetric in *h* and *e* (Good, 1966, 1968). Hence, Good identifies the degree to which *h* weakly explains *e* [see Eq. (3)] with the information concerning *h* provided by *e* or equivalently, and perhaps more appropriately, the information concerning *e* provided by *h*.

Since Inf(*e*) is a decreasing function of *P*(*e*), it could be taken to represent the degree to which *e* is surprising, in which case Inf$(e\vert h)$ would represent the degree to which *e* is surprising given *h*. Good’s weak measure of explanatory power can then be understood as representing how well *h* reduces the degree to which *e* is found to be surprising since it can be expressed as follows:^13^

14 $$\begin{aligned} {\mathcal {E}}_3(e,h) = \text{ log } \left[ \frac{P(e\vert h)}{P(e)} \right] = \text{ Inf }(e) - \text{ Inf }(e\vert h). \end{aligned}$$

Schupbach and Sprenger (2011) also interpret their measure of explanatory power in terms of reducing surprise, but there are a couple of advantages to Good’s measure in this respect. First, as we have just seen, Good’s weak measure can be formulated very straightforwardly in terms of semantic information.

Second, Schupbach and Sprenger’s measure, ${\mathcal {E}}_1$, fails to discriminate appropriately in terms of reduction of surprise for different explananda which are entailed by a hypothesis, and the same is true of Crupi and Tentori’s measure, ${\mathcal {E}}_2$, since both give the maximum value of one in such cases. Suppose that $e_1$ is very surprising in light of background knowledge, while $e_2$ is not surprising at all. Further suppose that *h* entails $e_1$ and also $e_2$. While ${\mathcal {E}}_1$ and ${\mathcal {E}}_2$ quantify the degree to which *h* explains $e_1$ to be the same as the degree to which it explains $e_2$, according to Good’s measure, ${\mathcal {E}}_3$, *h* provides a much better weak explanation of $e_1$ than it does of $e_2$. In fact, since Inf$(e_1\vert h) =$ Inf$(e_2\vert h) = 0$, the degree to which *h* explains $e_1$ is just Inf$(e_1)$ and similarly the degree to which *h* explains $e_2$ is Inf$(e_2)$ according to ${\mathcal {E}}_3$. Since Inf$(e_1)$ can be thought of as the degree to which $e_1$ is surprising in light of background knowledge only, it is clearly much greater than Inf$(e_2)$. As noted earlier, ${\mathcal {E}}_1$ and ${\mathcal {E}}_2$ are better thought of as measures of the degree to which *h* entails *e*.

Good’s strong measure of explanatory power, ${\mathcal {E}}_5$ [see Eq. (5)], can be expressed in terms of semantic information as follows:

15 $$\begin{aligned} {\mathcal {E}}_5(e,h)= & {} \text{ log } \left( \frac{P(e\vert h)}{P(e)} \right) + \gamma \text{ log } P(h) \nonumber \\= & {} \text{ Inf }(e) - \text{ Inf }(e\vert h) - \gamma \text{ Inf }(h) \nonumber \\= & {} \text{ Inf }(e,h) - \gamma \text{ Inf }(h). \end{aligned}$$

In light of our discussion, we can then say that strong explanatory power measures how well *h* reduces the degree to which *e* is found surprising together with the inclusion of a penalty for the complexity of *h*.

### Making Good’s measure precise

Recall that Good’s measure, ${\mathcal {E}}_5$, has a parameter, $\gamma$, which is required to be in the interval (0, 1). Can a particular value for $\gamma$ be defended? As Good pointed out, ${\mathcal {E}}_5$ can be expressed as follows:

16 $$\begin{aligned} {\mathcal {E}}_5(e,h) = (1 - \gamma ) \text {Inf}(h,e) - \gamma \text {Inf}(h\vert e). \end{aligned}$$

On the basis of this expression, he suggested $\gamma = \nicefrac {1}{2}$ as the simplest explicatum of ${\mathcal {E}}_5$ since it gives equal weighting to (weak) explanatory power and the term $-\text {Inf}(h\vert e)$, which he associates with ‘the avoidance of “clutter”’. However, while Good’s suggestion is not implausible a more convincing justification is needed.

To address this point, we can draw on a complexity criterion proposed for explanatory goodness (Glass, 2023). The criterion requires that for an explanation *h* of explanandum *e* to be a good one, the reduction in complexity of *e* brought about by *h* must be greater than the complexity introduced by *h* in the context of *e*, where the first of these quantities is represented by $\text {Inf}(h,e)$ and the second by $\text {Inf}(h\vert e)$. Expressed in terms of strong explanatory power, it is:

Complexity criterion for strong explanatory power. If ${\mathcal {E}}_S(e,h)$ is a measure of strong explanatory power of *h* for *e* then:

17 $$\begin{aligned} {\mathcal {E}}_S(e,h) \gtreqless 0 \;\; \text {if and only if} \;\; \text {Inf}(h,e) \gtreqless \text {Inf}(h\vert e). \end{aligned}$$

Note that since $\text {Inf}(e,h)$ is Good’s weak measure and $\text {Inf}(h\vert e) \ge 0$, this means that a positive value of weak explanatory power is necessary, but not sufficient, for an explanation to be a good one.

In light of (16), ${\mathcal {E}}_5(h,e) > 0$ if and only if $(1 - \gamma ) \text {Inf}(h,e) > \gamma \text {Inf}(h\vert e)$ and hence if ${\mathcal {E}}_5$ is to satisfy the complexity criterion, $\gamma$ must be $\nicefrac {1}{2}$. This provides a strong justification for adopting this specific version of Good’s measure and, as noted earlier, for a given explanandum *e* this will give the same ordering of hypotheses as measure ${\mathcal {E}}_6$.

Let us now return to example from Sect. 2.4 about a bag containing 99 fair coins and one with an objective chance of 0.51 of landing heads. On being tossed, a randomly selected coin lands heads (*e*) and we considered the hypotheses ‘the selected coin is fair’ ($h_1$) and ‘the selected coin is biased’ ($h_2$). Using Good’s measure with $\gamma = \nicefrac {1}{2}$, we find that ${\mathcal {E}}_5(e,h_1) \simeq \text {log}(0.9998) + \text {log}(0.9950) \simeq -0.0023$ which is greater than ${\mathcal {E}}_5(e,h_2) \simeq \text {log}(1.0198) + \text {log}(0.1) \simeq -0.9915$. So $h_1$ is indeed the better explanation according to ${\mathcal {E}}_5$, whereas $h_2$ would be judged better by weak measures since it is positively relevant to *e* while $h_1$ is not. According to ${\mathcal {E}}_5$, $h_2$ does not sufficiently reduce the complexity of *e* to compensate for the complexity introduced by $h_2$. Notice, however, that even though ${\mathcal {E}}_5$ judges $h_1$ to be better, it is clearly deficient in the sense that it has a negative degree of explanatory power, so it might be more accurate to say that $h_1$ is not as bad an explanation as $h_2$.

### Explanatory virtues and inference to the best explanation

We have already seen that Good relates his strong measure of explanatory power to the explanatory virtue of simplicity. According to his version of Ockham’s razor, if two hypotheses have equal likelihoods with respect to the explanandum we should prefer the simpler of the two, which he says is ‘equivalent to the choice of the more probable hypothesis’ (1968, p. 139). Given the discussion in Sects. 2.2 and 2.3, Good’s measure does indeed accommodate simplicity in a way that weak measures do not.

Good’s measure is also able to do justice to other explanatory virtues such as scope and unification. More specifically, it is his weak measure, which is a factor in his strong measure, that is able to capture these virtues. In terms of explanatory scope, we have already seen from Eq. (10) that the explanatory power of a hypothesis increases as it explains more evidence. In terms of unification, Myrvold (2003) develops an account in terms of informational relevance. Expressing a result of Myrvold’s in terms of Good’s measure of weak explanatory power gives:

18 $$\begin{aligned} {\mathcal {E}}_3(e_1 \wedge e_2,h) = {\mathcal {E}}_3(e_1,h) + {\mathcal {E}}_3(e_2,h) + U(e_1,e_2;h), \end{aligned}$$

where $U(e_1,e_2;h)$ is the degree to which *h* unifies $e_1$ and $e_2$ and is given by

19 $$\begin{aligned} U(e_1,e_2;h) = I(e_1,e_2\vert h) - I(e_1,e_2). \end{aligned}$$

hence *h* weakly explains $e_1 \wedge e_2$ to a degree that is the sum of how well it weakly explains $e_1$ and $e_2$ separately plus the degree to which it unifies them. It follows that if the sum of the weak explanatory power for $e_1$ and $e_2$ is the same for two hypotheses, then the one that unifies $e_1$ and $e_2$ more will have greater weak explanatory power. If they also have the same priors, then the hypothesis that unifies $e_1$ and $e_2$ more will have greater strong explanatory power as well. A similar conclusion can be reached concerning Whewell’s (1847) ‘consilience of inductions’ in terms of the value of diverse evidence (see, McGrew, 2016).

Does this mean that Good’s strong measure fully captures explanatory goodness? The various weak measures may well capture an aspect of explanatory goodness, but since they fail to accommodate simplicity, I have argued that they are not plausible candidates of explanatory goodness in a general sense. Since Good’s measure incorporates simplicity as well as the other virtues described above, it is a much more plausible candidate. Whether it fully captures explanatory goodness, however, is another matter. As acknowledged in Sect. 2.1, there may be some limitations to what can be captured probabilistically and this could include limitations arising from the fact that the account does not attempt to capture what constitutes an explanation. Also, the current approach does not take into account the potential relevance of manipulations to explanatory goodness. Eva and Stern (2019) have shown how this can be done for Schupbach and Sprenger’s measure of explanatory power, so it would be interesting to explore whether a similar approach might be appropriate for the current measure. Nevertheless, as it stands, Good’s measure does seem to go a long way to capturing key aspects of explanatory goodness.

A related topic concerns the relevance of Good’s strong measure to IBE. Recent work has demonstrated the merits of ${\mathcal {E}}_6$ [Eq. (6)] in this regard (Glass, 2021) and, as we have seen, it produces the same ranking as Good’s strong measure when $\gamma$ is $\nicefrac {1}{2}$. Results showed that using this measure for IBE finds the actual or true hypothesis much more frequently than versions of IBE based on weak measures. There is a lot more that could be said about explanatory virtues and IBE, but this brief discussion suggests that Good’s strong measure does well on both fronts.

## Conclusion

Strong measures of explanatory power attempt to strike a balance between how well a hypothesis accounts for the explanandum (weak explanatory power) and the improbability/complexity of the hypothesis. As such, they can be viewed as ways of making Ockham’s razor precise. While weak measures seek to capture an important aspect of explanation, I have argued that strong measures are better for quantifying explanatory goodness. In defence of Good’s strong measure, I have presented two new derivations of it, explored its connection with information theory and explanatory virtues, shown how it can be made precise, and addressed objections to it. Since Good’s strong measure depends on his weak measure, I have also presented several reasons for preferring his weak measure to the other weak measures. In particular, his weak measure is able to differentiate between explanatory power in cases where a given hypothesis entails two explananda where one is more surprising than the other.

There are various directions for further work. As noted above, it would be interesting to explore the potential relevance of manipulations to explanatory goodness in the context of Good’s measure. Also, in debates about IBE and Bayesianism, it is usually assumed that a Bayesian approach requires selecting the hypothesis with the highest posterior probability. However, this is not the case for the Bayesian approach to IBE based on Good’s measure. This is particularly relevant in cases where there are multiple compatible hypotheses. Strong measures of explanatory power should shed light on when it is appropriate to accept conjunctive explanations involving two or more hypotheses rather than just a single hypothesis.

## Appendix

### Proof of theorem 1

#### Lemma A.1

If $e^* \in L_c$ is probabilistically independent of *e*, $h \in L_c$ and their conjunction, then ${\mathcal {E}}_W(e \wedge e^*, h) = {\mathcal {E}}_W(e, h)$.

#### Proof

Let *e*, *h*, $e^* \in L_c$ and $P \in {{\varvec{P}}}$ be such that $e^*$ is independent of *e*, *h* and their conjunction according to *P*.

Suppose also that $e_2, h_2 \in L_c$. We can construct a probability distribution $P' \in {{\varvec{P}}}$ such that $P'(\pm e \wedge \pm h \wedge \pm e^*) = P(\pm e \wedge \pm h \wedge \pm e^*)$, where $\pm p$ denotes either *p* or $\lnot p$, so that all probabilities involving logical combinations of *e*, *h* and $e^*$ are preserved.

Now we can specify $P'$ in such a way that each of $e_2$ and $h_2$ is independent of *e*, *h*, $e\wedge h$ and $e^*$, and $e_2$ and $h_2$ are also independent of each other. To obtain such a distribution we can set conditional probabilities as follows:

(i) $$P'(e_2\vert \pm e \wedge \pm h \wedge \pm e^*) = a \in (0,1)$$,
(ii) $$P'(h_2\vert \pm e \wedge \pm h \wedge \pm e^* \wedge \pm e_2) = b \in (0,1)$$.

Note that $h_2$ is probabilistically independent of *e*, *h* and $h \wedge e$, as is $e^* \wedge e_2$ since a) $P'(e^* \wedge e_2\vert e) = P'(e_2\vert e \wedge e^*)P'(e^*\vert e) = P'(e_2\vert e^*)P'(e^*) = P'(e^* \wedge e_2)$ and so $e^* \wedge e_2$ is independent of *e*, b) $P'(e^* \wedge e_2\vert h) = P'(e_2\vert h \wedge e^*)P'(e^*\vert h) = P'(e_2\vert e^*)P'(e^*) = P'(e^* \wedge e_2)$ and so $e^* \wedge e_2$ is independent of *h*, and c) $P'(e^* \wedge e_2\vert h \wedge e) = P'(e_2\vert h \wedge e \wedge e^*)P'(e^*\vert h \wedge e) = P'(e_2\vert e^*)P'(e^*) = P'(e^* \wedge e_2)$ and so $e^* \wedge e_2$ is independent of $h \wedge e$. Hence, given (A3), the relevant conditions for applying (A4) as follows are satisfied^14^

$$\begin{aligned} {\mathcal {E}}_W(e \wedge e^* \wedge e_2, h \wedge h_2)= & {} w_c[{\mathcal {E}}_W(e, h),{\mathcal {E}}_W(e^* \wedge e_2, h_2)] \\= & {} w_c[{\mathcal {E}}_W(e, h),\alpha ]. \end{aligned}$$

Similarly, we can show that the relevant conditions for applying (A4) as below are also satisfied

$$\begin{aligned} {\mathcal {E}}_W(e \wedge e^* \wedge e_2, h \wedge h_2)= & {} w_c[{\mathcal {E}}_W(e \wedge e^*, h),{\mathcal {E}}_W(e_2, h_2)] \\= & {} w_c[{\mathcal {E}}_W(e \wedge e^*, h),\alpha ]. \end{aligned}$$

Since $w_c$ is strictly increasing in each argument, it follows that ${\mathcal {E}}_W(e \wedge e^*, h) = {\mathcal {E}}_W(e, h)$. This holds for distribution $P'$, but since it was constructed so as to preserve the probabilities for all logical combinations of *e*, *h* and $e^*$, it also holds in distribution *P*. This establishes lemma A.1. $\square$

Lemma A.1 will be used later in the proof of theorem 1 (in the proof of lemma A.3), but first it will be useful to introduce another lemma. Note that from (A1) it follows that there exist continuous, differentiable functions $w_1$ and $s_1$ such that for any *e*, $h \in L_c$ and any $P \in {{\varvec{P}}}$, ${\mathcal {E}}_W(e,h) = w_1[P(h\vert e),P(h),P(e)]$ and ${\mathcal {E}}_S(e,h) = s_1[P(h\vert e),P(h),P(e)]$.

To simplify matters we can identify triplets (*x*, *y*, *z*) representing $[P(h\vert e),P(h),P(e)]$ that satisfy the following conditions:^15^

1. $$0< y,z < 1$$
2. $$0 \le x \le 1$$
3. $$x \ge \frac{y+z-1}{z}$$ since $$xz = P(e \wedge h) \ge P(e) + P(h) - 1 = y + z - 1$$.
4. $$x \le y/z$$ since $$xz = P(e \wedge h) \le P(h) = y$$.

Let us then posit $w_1: \{(x,y,z)\in [0,1]\times (0,1)^2 \; \vert \; \frac{y}{z} \le x \le \frac{y+z-1}{z}\} \rightarrow {\mathbb {R}}$ and denote the domain of $w_1$ as $D_{w_1}$.

#### Lemma A.2

For any $x,y,z_1, z_2$ such that $x \in [0,1]$, $y,z_1, z_2 \in (0,1)$ and $\frac{y}{z_1} \le x \le \frac{y+z_1-1}{z_1}$ and $\frac{y}{z_2} \le x \le \frac{y+z_2-1}{z_2}$, there exist $e, e^*, h \in L_c$ and $P \in {{\varvec{P}}}$ such that $P(h\vert e) = P(h\vert e \wedge e^*) = x$, $P(h) = y$, $P(e) = z_1$ and $P(e \wedge e^*) = z_2$ where $P(e^*) = z_2/z_1$ and so $P(e \wedge e^*) = P(e)P(e^*)$.

#### Proof

This can be achieved by means of the following probability assignments:

- $$P(h \wedge e \wedge e^*) = xz_2$$,
- $$P(h \wedge e \wedge \lnot e^*) = x(z_1 - z_2)$$,
- $$P(h \wedge \lnot e \wedge e^*) = (y - xz_1)z_2/z_1$$,
- $$P(h \wedge \lnot e \wedge \lnot e^*) = (y - xz_1)(1 - z_2/z_1)$$,
- $$P(\lnot h \wedge e \wedge e^*) = (1 - x)z_2$$,
- $$P(\lnot h \wedge e \wedge \lnot e^*) = (1 - x)(z_1 - z_2)$$,
- $$P(\lnot h \wedge \lnot e \wedge e^*) = [(1 - y) - (1 - x)z_1]z_2/z_1$$,
- $$P(\lnot h \wedge \lnot e \wedge \lnot e^*) = [(1 - y) - (1 - x)z_1](1 - z_2/z_1)$$.

$$\square $$

#### Lemma A.3

There is a continuous, differentiable function $s_2$ such that for any *e*, $h \in L_c$ and any $P \in {{\varvec{P}}}$, ${\mathcal {E}}_S(e,h) = s_2[P(h\vert e),P(h)]$.

#### Proof

Suppose there exist $(x,y,z_1)$ and $(x,y,z_2) \in D_{w_1}$, the domain of $w_1$, such that $w_1(x,y,z_1) \ne w_1(x,y,z_2)$. Then, by lemma A.2 there exist $e, e^*, h \in L_c$ and $P \in {{\varvec{P}}}$ such that $P(h\vert e) = P(h\vert e \wedge e^*) = x$, $P(h) = y$, $P(e) = z_1$ and $P(e \wedge e^*) = z_2$ where $P(e^*) = z_2/z_1$. Clearly, $P(e \wedge e^*) = P(e)P(e^*)$ so $e^*$ is independent of *e*. Similarly, $P(h \wedge e^*) = xz_2 + (y-xz_1)z_2/z_1 = yz_2/z_1 = P(h)P(e^*)$ so $e^*$ is independent of *h*, and $P(h \wedge e \wedge e^*) = xz_2 = xz_1.z_2/z_1 = P(h \wedge e)P(e^*)$ so $e^*$ is also independent of $h \wedge e$. Thus, there exist $e, e^*, h \in L_c$ and $P \in {{\varvec{P}}}$ such that ${\mathcal {E}}_W(e, h) = w_1(x,y,z_1) \ne w_1(x,y,z_2) = {\mathcal {E}}_W(e \wedge e^*, h)$ even though $e^*$ is independent of *e*, *h* and their conjunction, contradicting lemma A.1. Conversely, lemma A.1 implies that for any $(x,y,z_1)$ and $(x,y,z_2) \in D_{w_1}$, $w_1(x,y,z_1) = w_1(x,y,z_2)$. Hence, lemma A.1 requires that there must exist $w_2$ such that, for any $e, h \in L_c$ and $P \in {{\varvec{P}}}$, ${\mathcal {E}}_W(e,h) = w_2[P(h\vert e),P(h)]$ and $w_2(x,y) = w_1(x,y,z)$. Hence it follows from (A2) that there is a differentiable function $s_2$ such that, for any $e, h \in L_c$ and $P \in {{\varvec{P}}}$, ${\mathcal {E}}_S(e,h) = s_2[P(h\vert e),P(h)]$ since a differentiable function of differentiable functions is itself differentiable. This establishes lemma A.3. $\square$

Given lemma A.3 and (A4), Good shows in his 1968 paper that, up to a differentiable monotonic transformation, ${\mathcal {E}}_S(e,h)$ is given by

A1 $$\begin{aligned} \textrm{log}[P(h\vert e)] + (\gamma - 1) \; \textrm{log}[P(h)], \end{aligned}$$

where $\gamma$ is a constant or alternatively,

A2 $$\begin{aligned} \textrm{log}\left[ \frac{P(e\vert h)}{P(e)}\right] + \gamma \; \textrm{log}[P(h)]. \end{aligned}$$

(A5) implies that if $P(e\vert h_1) = P(e\vert h_2)$, then $\gamma \; \textrm{log}[P(h_1)] \gtreqless \gamma \; \textrm{log}[P(h_2)]$ if and only if $P(h_1) \gtreqless P(h_2)$ and so $\gamma > 0$. (A6) implies that if $P(h_1\vert e) = P(h_2\vert e)$, then $(\gamma - 1) \; \textrm{log}[P(h_1)] \gtreqless (\gamma - 1) \; \textrm{log}[P(h_2)]$ if and only if $P(e\vert h_1) \gtreqless P(e\vert h_2)$, but since $P(h_1\vert e) = P(h_2\vert e)$ this will be the case if only if $P(h_1) \lesseqgtr P(h_2)$ and hence $\gamma < 1$.

These conditions also require that any monotonic transformation of this function must be increasing. Suppose that ${\mathcal {E}}_S(e,h)$ were a decreasing function of (A2). Suppose also that $P(e\vert h_1) = P(e\vert h_2)$. Then, if $\gamma \, \textrm{log}[P(h_1)] > \gamma \, \textrm{log}[P(h_2)]$, and hence $P(h_1) > P(h_2)$, it would follow that ${\mathcal {E}}_S(e,h_1) \le {\mathcal {E}}_S(e,h_2)$, which contradicts (A5). This establishes theorem 1. $\square$

### Proof of theorem 2

The result for ${\mathcal {E}}_W$ follows from (A1), (A7) and (A8) as demonstrated by theorem 3 of Cohen (2016), which was in turn proved in the context of ${\mathcal {E}}_3$ as a confirmation measure by Crupi et al. (2013). Lemma A.1 follows trivially given the result for ${\mathcal {E}}_W$ and lemma A.3 then follows straightforwardly from lemma A.1 and (A2). The result for ${\mathcal {E}}_S$ can then by established from the relevant part of the proof for theorem 1 based on lemma A.3, (A4), (A5) and (A6). $\square$

## Footnotes

1 This is clear from Crupi and Tentori’s expression ‘assuming candidate explanans *h*’.

2 Even if that is not the case, measures of explanatory power may still be applicable in cases where the relevant probabilities are accessible. It would be impossible to do justice to the vast literature on the problem of old evidence here, but for some recent proposals see Sprenger (2015) and Eva and Hartmann (2020).

3 Hitchcock (1999) discusses a similar example to motivate indeterministic contrastive explanation. He considers a person who is puzzled that a photon was transmitted rather than absorbed when the polarizer is believed to be aligned along the horizontal, but suggests that noting that the polarizer was in fact aligned very close to the vertical explains the contrast. For discussion of the relevance of high likelihoods in the context of inference to the best explanation, see Lipton (2004, Chap. 7). Finally, note that in the proposed approach, higher likelihood does not always result in greater explanatory goodness (see Sect. 2.4).

4 It could be questioned whether this does justice to our intuitions about complexity, but as we shall see in Sect. 4, it is based on a widely used account of semantic information and is relevant to some of the formal results presented. Given this approach, the simplicity of a hypothesis can be represented as the negative of its complexity.

5 This is Schupbach and Sprenger’s third adequacy condition, CA3. Note that two propositions *p* and *q* are said to be independent if $P(p \wedge q) = P(p)P(q)$ and they are conditionally independent given *r* if $P(p \wedge q \vert r) = P(p \vert r)P(q \vert r)$.

6 One response might be to deny that $h_1$ provides an explanation since it lowers the probability of *e* (for discussion of this topic, particularly in the context of causal explanation, see Salmon (1980), Hitchcock (2004)). In the current context, $h_1$ does seem to provide an explanation. It might be that $h_1$ is not a very good explanation; just better than $h_2$ (see Sect. 4.4). Note that measures ${\mathcal {E}}_1 - {\mathcal {E}}_4$ also allow for negative degrees of explanatory power.

7 A1 corresponds closely to one of Good’s axioms, while A2 corresponds to an assumption he states for a measure of strong explanatory power, as does the part of A4 that refers to ${\mathcal {E}}_S$. A5 and A6 do not correspond to Good’s formally stated axioms and assumptions, but they provide a formal way to constrain the form of ${\mathcal {E}}_5$ and more specifically the possible values of $\gamma$ instead of his informal discussion.

8 That is, ${\mathcal {E}}_W$ satisfies A1, A3 and A4, while ${\mathcal {E}}_S$ satisfies A1, A2, A4, A5 and A6.

9 Good’s weak measure turns out to have a significant advantage over other measures in terms of how it accounts for reduction or surprise as we shall see in Sect. 4.3.

10 Strictly speaking, ${\mathcal {E}}_W$ satisfies A1, A7 and A8 while ${\mathcal {E}}_S$ satisfies A1, A2, A4, A5 and A6. Note that only the part of A4 relating to ${\mathcal {E}}_S$ is required.

11 Both ${\mathcal {E}}_1$ and ${\mathcal {E}}_2$ are well-known measures of the degree to which *h* confirms *e* and can very plausibly be considered as measures of confirmation in the sense of partial entailment since they are maximal when *h* entails *e* and minimal when *h* entails the negation of *e*. For further discussion, see Fitelson (2006), Crupi and Tentori (2013).

12 ${\mathcal {E}}_6$ and ${\mathcal {E}}_7$ do not face the problems of irrelevant evidence, irrelevant conjunction or relevant evidence. ${\mathcal {E}}_6$ also satisfies A4, but ${\mathcal {E}}_7$ does not.

13 See Crupi and Tentori (2014) and Milne (2014) for similar discussions in the context of confirmation.
